# Incidence of traumatic dental injury in Valencia, Spain

**DOI:** 10.4317/medoral.23630

**Published:** 2020-05-10

**Authors:** Vicente Faus-Matoses, Ignacio Faus-Matoses, Celia Ruiz-Sánchez, María Faus-Damiá, Vicente José Faus-Llácer

**Affiliations:** 1 DDS, MSc, PhD. Co-director of the Master of Restorative Dentistry and Endodontics, Department of Stomatology, Medicine and Dental School, Valencia University, Spain; 2 DDS, MSc, PhD. Master in Orthodontics, Department of Stomatology, Faculty of Medicine and Dentistry, University of Valencia, Spain; 3 DDS, MSc. Master of Restorative Dentistry and Endodontics, Department of Stomatology, Faculty of Medicine and Dentistry, University of Valencia, Spain; 4 DDS, MSc, PhD. Master in Periodontics, Department of Stomatology, Faculty of Medicine and Dentistry, University of Valencia, Spain; 5 MD, DDS, PhD. Director of the Master of Restorative Dentistry and Endodontics, Department of Stomatology, Medicine and Dental School, Valencia University, Spain

## Abstract

**Background:**

While traumatic dental injuries (TDI) are an increasingly frequent occurrence in everyday dental practice, little research on TDIs has been published in Spain. The aim of this study was to determine the incidence of TDIs in a population in Valencia (Spain) and investigate influential variables. In addition, a protocol for TDI data collection is proposed.

**Material and Methods:**

This retrospective study compiled data from patients attending a private dental clinic between January 2003 and December 2014. The data were collected using a specially-elaborated protocol entitled “Emergency care of acute dental trauma”. Patients responded to each item, and data was added from case radiographs and photographs. Data were entered in a Microsoft Office Excel spreadsheet and submitted for analysis by SPSS 15.0 software (Chicago, IL) applying 2-way analysis of variance (ANOVA) (*p*<0.01).

**Results:**

481 TDIs in 251 patients were examined at a private dental practice in Valencia. The population comprised 62.5% men and 37.5% women, aged 1 to 78 years. The highest frequency of tooth injuries occurred in children aged 9 years or younger. The most frequent injury was non-complicated crown fracture (43.2%). Upper central incisors were the most commonly affected teeth. The most frequent place where TDI was produced was in the street (28.7%), tripping over an immobile object being the most common cause (29%).

**Conclusions:**

Thanks to the protocol elaborated for the purposes of this work, it was possible to compile a large quantity of data on TDI, facilitating future prevention and comparison with other regions. The results obtained concur with those published in the literature.

** Key words:**Dental trauma, traumatic dental injuries, prevalence, epidemiology, risk factors.

## Introduction

Traumatic dental injury (TDI) is a branch of dentistry that encompasses epidemiology, etiology, prevention, assessment, diagnosis and treatment of trauma produced in teeth, jaws, and the surrounding tissues (1). At the present time, TDIs are the second most common reason patients seek dental care after caries, and could become the primary cause in the future. Of course, TDIs are produced suddenly, immediately, and unexpectedly; their treatment and prevention are a recurrent topic in dental research (2).

The etiology of dental trauma has been extensively researched, the most common causes being falls, sports injures, traffic accidents and Fights. These causes may be aggravated by predisposing factors such as the practice of contact sports, malocclusions, and the time of year (3-5). However, there has been little epidemiological research into dental trauma in Spain. The scant research that has been carried out to date has not generated much data or proposed a reproducible protocol for data collection, a lack that the present study set out to address (6,7).

So, the aim of this study was to determine the distribution of TDI to deciduous and permanent teeth in a population in Valencia (Spain). Based on the existing literature, several variables were analyzed relating to dental trauma including gender, age, the most common types of lesion occurring in the population under investigation according to Andreasen’s classification, the most frequently affected teeth, the time elapsed before seeking dental attention following the trauma, the places where trauma most often occurs, etiology, Angle class, whether patients wear orthodontic apparatus, and the time of year when trauma occurs (3,4).

## Material and Methods

This retrospective cross-sectional study included all the patients presenting TDIs who attended a private clinic situated in the town of Algemesí (Valencia, Spain) between January 2003 and December 2014. The investigation was performed following guidelines established in the Declaration of Helsinki, whereby patients were required to sign a consent form after being provided with full information about the study.

**Figure 1:**
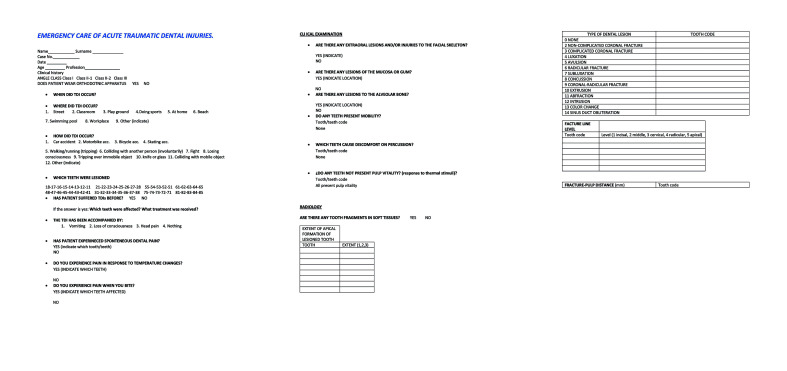
Protocol: “Emergency care of acute traumatic dental injuries”.

Cases of TDI were investigated on the basis of data obtained by means of a protocol entitled “Emergency care of acute traumatic dental injury” (Fig. 1), elaborated by modifying and developing standardized questionnaires drawn from various published articles (8-10). The protocol was completed during each patient’s initial visit to the clinic after suffering TDI.

Field work to fill out each protocol began by compiling each patient’s clinical medical and dental history, and performing extraoral and intraoral examinations. The pulp vitality of the affected teeth was checked by means of (refrigerant spray) thermal testing (ROEKO Endo Frost, Coltene, Whaledent, Switzerland) and electric testing (Electronic Pulp Tester, Sybron Endo). Responses to percussion and palpation were assessed, and the teeth that had suffered trauma were recorded. Afterwards, radiographic examination was performed using an orthopantomograph (Planmeca Promax®, Planmeca Oy, Helsinki, Finland) and periapical radiographs (Dígora®, Soredex, Tuusula, Finland) with an intra-oral X-ray system (Trophy© 2100 Intraoral X-Ray System 110V/170cm) and a Rinn type positioner (XCP BAi, Dentsply, Weybridge, UK), as well as cone beam computerized tomography (CBCT) when indicated. Lastly, each case was documented with photographs using a digital reflex camera (Nikon D90, Nikon© Corporation, Tokyo, Japan).

To complete the protocol, TDIs were classified according to the system recommended by Andreasen and teeth were identified according to the FDI system. Patient data was entered in a Microsoft Office Excel spreadsheet.

A bio-statistician with expertise in dentistry analyzed the data using SPSS 15.0 software (Chicago, IL, USA) applying 2-way analysis of variance (ANOVA). As a control measure, the non-parametric Kruskal-Wallis test, Mann-Whitney test and Pearson’s 2 test were applied. Statistical significance was set at a *P* value of below 1% (*p*<0.01).

## Results

The sample consisted of 251 patients who suffered some type of TDI, presenting a total of 481 affected teeth. Patients comprised 157 men (62.5%) and 94 women (37.5%), with an overall average age of 16 years, which ranged from one to 77 years. Statistically significant differences in traumatic dental injuries were found in relation to gender and age (*p*<0.01).

- Injury types

Among the 481 teeth that made up the sample, the most common types of injury were non-complicated coronal fracture, representing 43.2% of teeth affected by TDI, followed by 18.5% of teeth that did not suffer any type of traumatic lesion, and 12.1% that suffered luxation. The least frequent types of lesion were extrusion (2.3%), corono-radicular fracture (2.1%) and radicular fracture (1.7%) (Table 1).

- Teeth affected and associated lesions

**Figure 2:**
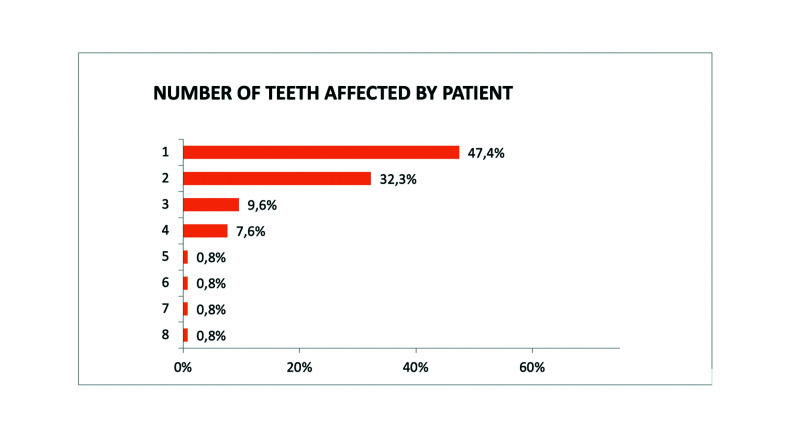
Number of teeth affected by patient.

The upper left central incisor was the most commonly affected tooth in permanent dentition (29.1%), while in deciduous teeth it was the upper right central incisor (4.6%). The number of affected teeth in a single patient ranged between one and eight. In 47.4% of patients, only one tooth was affected, 32.3% of the patients presented two traumatized teeth, and 9.6% had three (Fig. 2).

**Figure 3:**
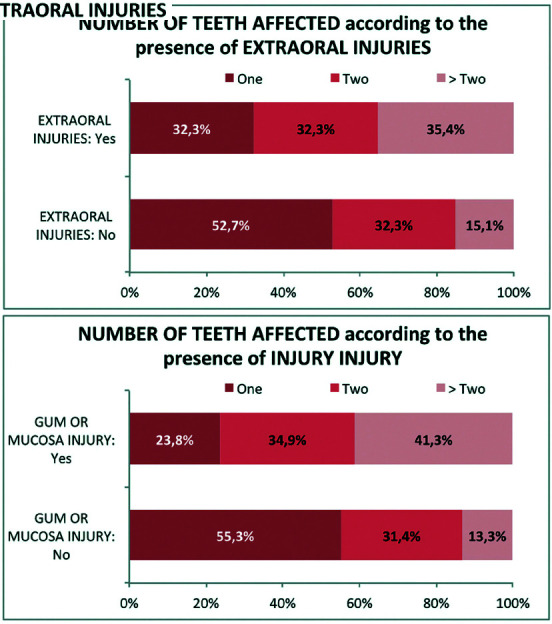
According to associated injuries - No OF TEETH AFFECTED

The number of affected teeth was significantly higher when patients suffered associated extraoral lesions or injures to the gums or mucosa than when they did not (*p*<0.01). The proportion of patients presenting more than two affected teeth was three times greater when a mucosal or gingival lesion was suffered, and double when there was an extraoral lesion present (Fig. 3).

**Table 1:**
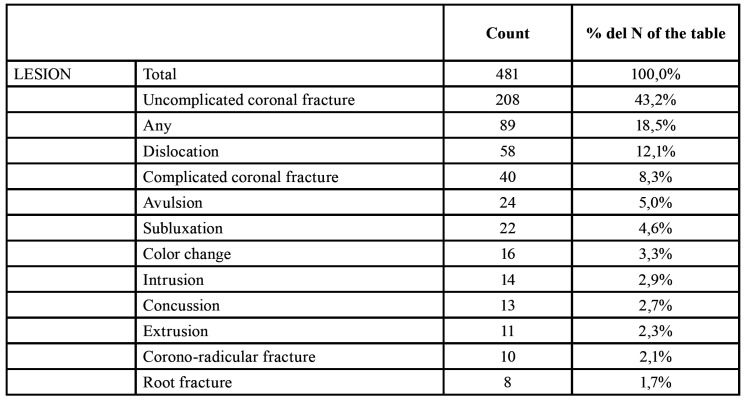
Lesion types.

- Time elapsed between trauma and treatment

The mean time elapsed between the moment of trauma and start of treatment was 3 days. Approximately one third (34.1%) of patients came to the clinic seeking treatment within 24 hours of the trauma incident, those who appeared later being the patients who suffered less severe TDIs. Spontaneous and thermal pains were more prevalent among patients who sought attention within 24 hours than among those who postponed coming for longer. Spontaneous and thermal pains occurred in 42% of teeth of those patients who attended the clinic within 24 hours, while the incidence of these types of pain decreased to 18-24% after the fifth day. Pain on biting had the same incidence until the fifth day without treatment, and thereafter decreased (Table 2).

**Table 2:**
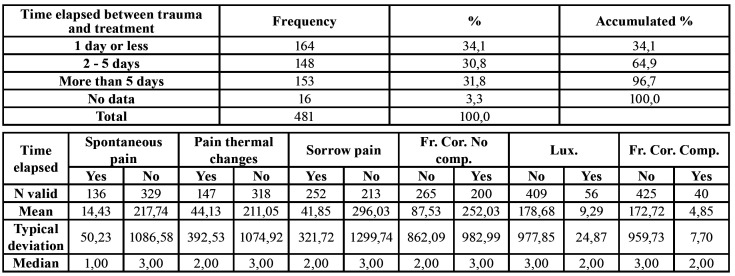
Time elapsed between moment of dental trauma and treatment in relation to PAIN and LESION TYPE.

- Place where TDI occurred and etiology

The most common place where TDIs occurred was in the street (28.7%), followed by the home (19.4%), the school playground (13.8%), and with less frequency the beach (5.3%), and the workplace (2%). The most common cause of TDI was tripping over an immobile object (29.0%), followed by tripping while walking (15.7%) (Table 3).

**Table 3:**
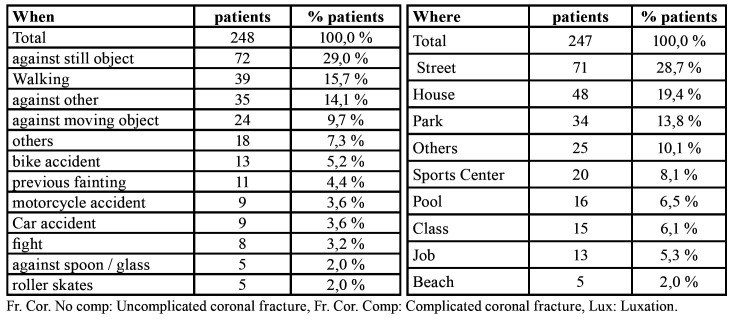
Place where TDI occurred and etiology.

- Angle class and orthodontic apparatus

The most common Angle class among patients was Class I (47.6%), followed by Class II-1 (46.2%), although no statistically significant difference was found between individuals presenting Class I and Class II-I. Patients wearing orthodontic apparatus did not suffer higher numbers of TDIs, in fact only 6.4% wore apparatus (Table 4).

**Table 4:**
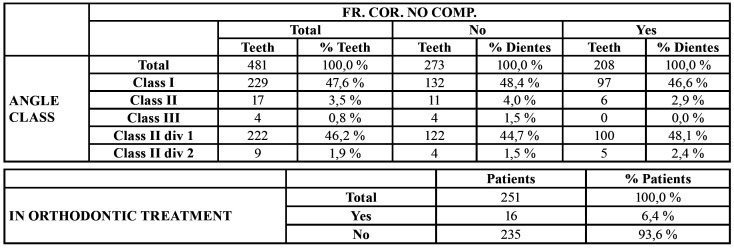
Angle class in relation to LESION TYPE. Patients undergoing orthodontic treatment. Distribution of TDI incidents according to time of year.

- Time of year

Most TDIs occurred during the autumn (28.2%), followed by the summer (25.2%), winter (24.8%), and in last place spring (21.8%). However, the proportion of DTIs by month or season did not show statistically significant differences (*p*>0.01).

## Discussion

In recent years, dentists have observed an increase in the prevalence of traumatic dental injuries (TDIs). An interdisciplinary approach is required to achieve good therapeutic results in cases of TDI, and this has become a topic of discussion among dental health professionals (1). The present study complied data derived from TDIs suffered by patients attending a private clinic in Valencia (Spain) between 2003 and 2014 in order to determine the incidence of this type of injury. In a society like ours, in which lifestyles and the social attitudes of children and adolescents have undergone considerable changes, it may be assumed that the incidence of these events will tend to rise in the future, and the etiological factors of TDI will alter (11).

According to the present work, males suffered more TDIs than females, a finding that agrees with other studies published in both Spain (6,12,13) and abroad (14,15). The mean age when TDIs were suffered was 16 years, up to the age of 9 being the age group when TDIs are suffered with greater frequency, which could be due to the fact that children are more active at this time of life (14,16). Although large differences between the sexes were not found as patient age increases, we believe that further research would confirm the hypothesis put forward by Glendor *et al*. who attribute the frequency of TDIs to types of activity rather than sex per se (17).

In agreement with most of the published literature, the upper central incisors suffer more lesions resulting from TDI (12,13,18,19). In general, more TDIs are produced to maxillary than mandibular teeth, a fact that could be due the protrusion and overjet of upper teeth, and to lip incompetence (20). Within this type of lesion, the most common were non-complicated enamel fractures, a finding that agrees with many other studies (18,20). According to Tzigkounakis, the teeth most commonly affected by avulsion are the upper central incisors (19), a result that concurs with the present study, in which all avulsed teeth were upper central incisors (3.3%).

There is no established system for classifying the causes of TDIs, and so they can only be described in terms of individual circumstances. In the present study, most TDIs occurred in the street, followed by those produced at home and at school, data that concur with other reports (18). According to the literature, recent years have seen an increase in TDIs resulting from traffic accidents (15), although this claim does not agree with our findings, in which moped, car or bicycle accidents where the least common causes of TDI.

The mean time elapsed between the moment of trauma and the start of treatment was more than 5 days, but this mean was distorted by a number of extreme values, the median being 3 days. This could be due to the patients not suffering any symptom or pain or to their not knowing what action to take to deal with the TDI. It should be noted that 34.1% of the patients came to the clinic within the first 24 hours after the incident that caused the TDI, a fact that coincides with other research (21,22).

In some studies, seasonal variations were found to be significant, summer and autumn being the seasons when TDIs are more commonly produced (23). However, the present study did not find statistically significant differences between either months or seasons, which could be due to the climate and the number of hours of sunshine in this part of the world.

The protocol elaborated for the purposes of the present work facilitated data collection and management. It could prove a useful resource in TDI prevention and management, and facilitate future comparison of the present findings with other regions. As the frequency of TDIs is on the increase, it would be useful to develop approaches to prevention and to provide more information about the effects and management of TDI to parents and teachers, and to recommend the use of mouth guards to groups at risk (17,18,20). Nevertheless, the present study did not provide novel information as the sample consisted of a sector of the population made up of patients who sought attention for TDI rather than a general population of patients in which it would be possible to determine how many had suffered TDIs and how many not.

## Conclusions

Analyzing the results obtained, it may be concluded that age, gender, Angle class, and the time of years are predisposing factors for dental trauma injury, as reported in numerous other articles.
